# Adenosine receptor signalling in Alzheimer’s disease

**DOI:** 10.1007/s11302-022-09883-1

**Published:** 2022-07-23

**Authors:** Phuc N. H. Trinh, Jo-Anne Baltos, Shane D. Hellyer, Lauren T. May, Karen J. Gregory

**Affiliations:** 1 Drug Discovery Biology, Monash Institute of Pharmaceutical Sciences, Monash University, Parkville, VIC 3052 Australia; 2 Department of Pharmacology, Monash University, Parkville, VIC 3052 Australia; 3 ARC Centre for Cryo-Electron Microscopy of Membrane Proteins, Monash Institute of Pharmaceutical Sciences, Parkville, 3052 Australia

**Keywords:** Adenosine, Dementia, Alzheimer’s, GPCR, Allosterism, Oligomerisation

## Abstract

Alzheimer’s disease (AD) is the most common dementia in the elderly and its increasing prevalence presents treatment challenges. Despite a better understanding of the disease, the current mainstay of treatment cannot modify pathogenesis or effectively address the associated cognitive and memory deficits. Emerging evidence suggests adenosine G protein-coupled receptors (GPCRs) are promising therapeutic targets for Alzheimer’s disease. The adenosine A_1_ and A_2A_ receptors are expressed in the human brain and have a proposed involvement in the pathogenesis of dementia. Targeting these receptors preclinically can mitigate pathogenic β-amyloid and tau neurotoxicity whilst improving cognition and memory. In this review, we provide an accessible summary of the literature on Alzheimer’s disease and the therapeutic potential of A_1_ and A_2A_ receptors. Although there are no available medicines targeting these receptors approved for treating dementia, we provide insights into some novel strategies, including allosterism and the targeting of oligomers, which may increase drug discovery success and enhance the therapeutic response.

## Introduction

Dementias are the most prevalent neurological disorder in the aged population, affecting more than 50 million people worldwide in 2020 [1]. In the absence of effective therapies, the number of people with dementia is projected to double every 20 years, reaching 152 million in 2050 [1]. While there are hundreds of described neurodegenerative dementias, the most common is AD [2]. Sporadic AD, the most common form, seems to be multifactorial, with age, genetic, and environmental factors contributing to disease risk, manifestation, and progression [3, 4]. AD is classically defined as a dual clinicopathological entity, meaning that to fully diagnose AD requires (i) observations of a specified clinical presentation over time, related to episodic memory impairment and other cognitive, behavioural, and neuropsychiatric abnormalities and (ii) observations of specific neurological changes (e.g. neurofibrillary tangles, amyloid plaques, synaptic loss). Recent advances in amyloid positron emission tomography (PET) and structural magnetic resonance imaging (MRI) have enabled the detection of in vivo biological evidence of AD pathologies to assist the classification of presymptomatic AD stages [5–7].

## Neuropathological alterations in Alzheimer’s disease

Compared to age-matched non-AD individuals, AD patients experience an accelerated rate of brain mass shrinkage due to neuronal death and grey matter loss; the amount of neuronal atrophy correlates with AD severity [8–11]. AD neuropathology is characterised by significant gyral shrinkage, widened sulci, and enlarged ventricles present in multiple areas within the brain, along with a significant reduction in the volume and/or cortical thickness of regions such as hippocampus and entorhinal, temporal, parietal, and frontal cortex [12–15]. There are currently three main theories regarding the mechanisms underlying neurodegeneration in AD. Although some features are shared by all three theories, each postulates a different causal event or sequence of events leading to neurodegeneration.

The amyloid cascade hypothesis posits the accumulation of toxic amyloid β (Aβ) peptides as the main cause of neurodegeneration [16]. The formation of amyloid plaques is driven by aberrant processing of amyloid precursor protein (APP) into Aβ40 and Aβ42 peptides, which normally play important physiological roles, including synaptic function, neuronal development and plasticity, and lipid homeostasis [17–19]. Although Aβ40 is more abundant than Aβ42 in normal conditions, in AD the ratio shifts in favour of Aβ42 generation. The formation of Aβ deposits could be due to altered Aβ42/Aβ40 ratios or the failure of Aβ clearance processes [20, 21]. Neuritic dense core Aβ plaques are deposited in the hippocampus, amygdala, and cortex, resulting in a cascade of events that include sustained inflammatory responses, imbalanced neuronal ionic homeostasis, altered kinase and phosphatase activity, tau phosphorylation, neurofibrillary tangle (NFT) formation, and neuronal and synapse loss [22–24]. Aβ pathology is widespread in early diseases, whereas tau pathology develops much later, suggesting changes in Aβ are the initial insult driving tau pathology. As such, the amyloid theory has become the dominant model of AD pathogenesis; guiding the development of potential AD disease-modifying treatments.

**Fig. 1:**
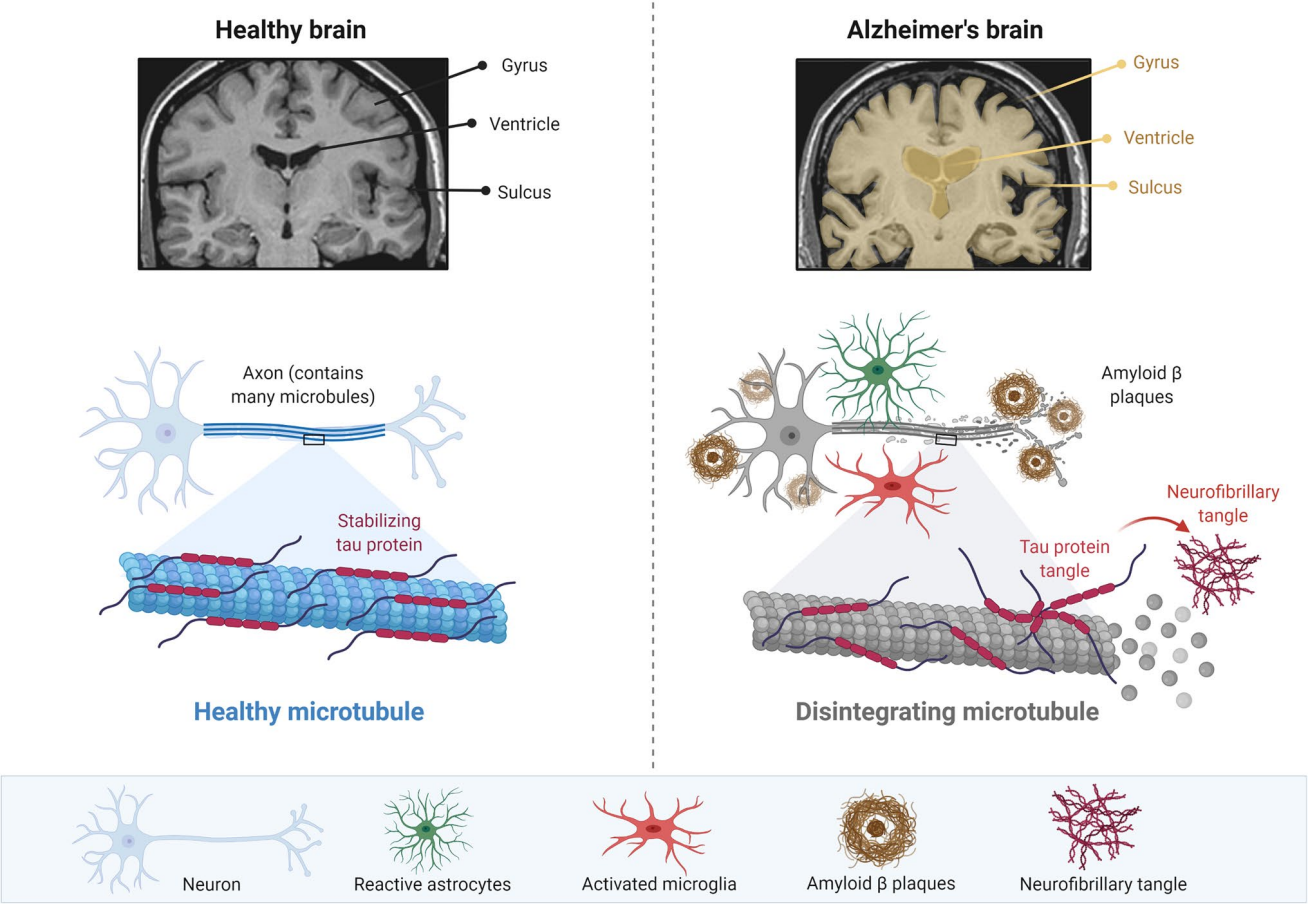
Schematic diagram of brain atrophy and neuropathological alterations between normal, healthy brain versus AD brain. On the left is a healthy aged brain, while on the right, an aged Alzheimer’s brain (shaded in yellow) has marked atrophy, including widened sulci, enlarged ventricles, and gyral shrinkage. At the cellular level, dying neurons are surrounded by reactive astrocytes, activated microglia, extracellular Aβ plaques, and intracellular neurofibrillary tangles in aged AD brain. Adapted from “Pathology of Alzheimer’s Disease 2” template by BioRender.com (2022). Retrieved from https://app.biorender.com/biorender-templates

**Fig. 2:**
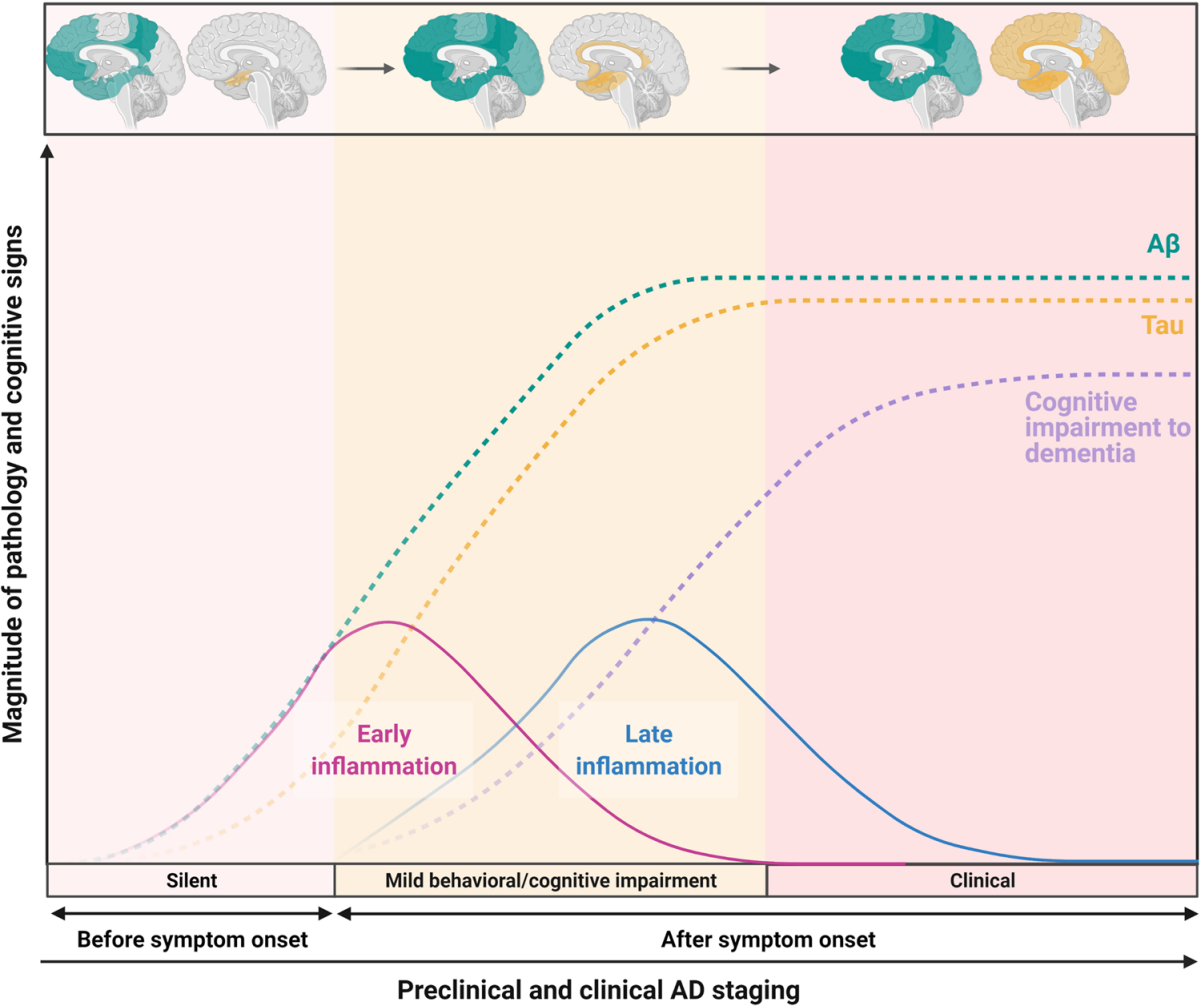
Simplified overview showing the spatiotemporal patterns of Aβ and tau pathologies in conjunction with proposed model of early and late inflammation occurrence during AD progression. Yellow and green shading in the brain indicates areas affected by tau and Aβ pathology, respectively. Extensive Aβ pathology can be detected in individuals with preclinical AD, and the extent of Aβ pathology alters minimally in symptomatic stages, whereas tau pathology develops considerably later. Further, early inflammation is likely to begin before the presence of Aβ plaques, while late inflammation should commence when the first Aβ plaques are established. The early and late inflammation overlaps at the later stage of preclinical AD. At the clinical stage, the late inflammation becomes predominant. The scheme of the evolution of the AD pathology is adapted from [304]. Created with BioRender.com

An opposing view suggests tau pathology is responsible for the initiation of AD neurodegeneration. Under normal conditions, soluble tau protein acts as a microtubule stabiliser, maintaining neuronal integrity, neurite outgrowth, and axonal transport [25–27]. The degree of tau phosphorylation is critical in regulating microtubule assembly and, in AD, tau protein becomes hyperphosphorylated through the actions of multiple kinases [28–34]. Hyperphosphorylated tau detaches from microtubules and self-aggregates to form NFTs, which disrupt axonal transport and subsequently lead to neuronal loss [35, 36] (Fig. 1). Furthermore, NFTs are often associated with Aβ plaques and chronic inflammation, as evidenced by the accumulation of activated astrocytes and microglia [37–41]. The tau theory is supported by the correlation between tau pathology and the degree of AD dementia, with the distribution and amount of NFTs in AD brains related to the severity and time course of the disease [42, 43]. In symptomatic subjects, NFTs are widespread and correlate well with the AD-affected functional brain circuits [44–47]. Importantly, Aβ and tau pathologies do not develop in the same brain region; thus, it is debatable whether the Aβ pathology drives tau pathology (Fig. 2). Targeting tau pathology with drugs or vaccines to inhibit tau aggregation or promote tau degradation is being investigated for both symptomatic and preventative treatment [48, 49].

Another pathological feature shared by all AD pathogenesis theories is chronic inflammation. The inflammation theory of AD pathogenesis postulates that activated microglia and reactive astrocytes trigger AD pathogenesis and this event precedes the presence of Aβ plaques and NFTs [50]. This theory is supported by the presence of inflammatory changes very early in AD neuropathology [51, 52] (Fig. 2). Given activated microglia and astrocytes are commonly found in close proximity to Aβ and tau deposits, dysfunction of astrocytes or microglia may lead to the formation of plaques and NFTs [53–57]. While the precise mechanisms are yet to be defined, it is suggested intracellular accumulation of Aβ oligomers leads to preclinical AD inflammation before clinical hallmarks of AD are present [58, 59]. The imbalance between pro-inflammatory and anti-inflammatory mechanisms and a shift from neuroprotective to neurotoxic glial phenotypes further exacerbates disease pathology, leading to late clinical AD inflammation [60–62] (Fig. 2). While the concept of chronic inflammation has been recognised as an important AD feature, most drug discovery efforts are directed towards agents targeting Aβ peptides and tau protein. This is likely due to an incomplete understanding of mechanisms underlying microglial and astrocytic activation and the mechanistic link between amyloid, tau, and inflammatory pathologies. Despite these unresolved problems, reducing neuroinflammation has recently attracted more interest and is still under investigation [63, 64].

## Glial cells in Alzheimer’s disease-associated inflammation

Microglia and astrocytes are two of the most common glial cells present in the CNS. Microglia are actively involved in regulating various aspects of neuroplasticity and mediating neuroprotection, while the principal role of astrocytes is to maintain overall brain homeostasis via the uptake and release of ions and neurotransmitters from the extracellular fluid surrounding neurons [65–69]. When the brain is injured, microglia and astrocytes undergo rapid activation, resulting in phagocytosis of cellular debris and production of neurotoxic and inflammatory compounds (e.g. reactive oxygen species, growth factors and cytokines) [66, 70]. In AD, chronic activation of glial cells results in the release of a variety of pro-inflammatory cytokines, chemokines, reactive oxygen species, and N-terminally truncated Aβs that impair neuronal activity and survival [71–75]. Analysis of neuroinflammatory gene expression in the frontal cortex of early-, mid-, and late-stage AD patients revealed activated microglia are present throughout the disease course. Additionally, there are signs of reactive astrogliosis in plaque-containing areas, with glial Aβ clearance mechanisms becoming impaired [76–78]. A buildup of Aβ oligomers and fibrils can activate microglial cell-surface receptors, leading to chronic activation of store-operated calcium (Ca^2+^) entry and upregulation of pro-inflammatory mediators [79–84]. Activated microglia and astrocytes intensify and sustain activation of each other via secretion of pro-inflammatory molecules, exacerbating chronic neuroinflammation and impairing their ability to promote neuronal survival, growth, synaptogenesis, and phagocytosis [85–88]. The best approach for anti-inflammatory use in AD is to target the activated microglia and/or reactive astrocytes to disrupt inflammation in the initial stages of AD. Indeed, there is increased interest in the expression and distribution of various GPCRs connected to microglial and astroglial activation in the AD brain and the ability to attenuate AD inflammation [89, 90].

## Current status of drug development

Pharmacotherapeutic options for patients diagnosed with AD are extremely limited, most only treat symptoms rather than disease progression. As such, it is imperative to seek new AD treatments to prevent, delay, and treat AD clinical symptoms. To date, only five agents have been approved based on modest symptomatic clinical effects; the cholinesterase inhibitors tacrine, donepezil, rivastigmine, and galantamine for mild-to-moderate AD and the glutamate antagonist memantine for moderate-to-severe AD [4]. Around 20% of ongoing clinical trials are focused on new symptomatic agents aimed at enhancing cognitive function through modulation of neurotransmitter synthesis, receptor activation and reuptake [91–93]. For disease-modifying treatments, attention for both Aβ-targeted and tau-related therapies has increased; as of March 2022, around thirty anti-Aβ or anti-tau agents are in phase 2 or 3 trials, with many more in the preclinical stages. However, there is an increasing diversity of targets for disease-modifying therapies, including vasculature, inflammation, and metabolism, reflecting the constantly changing understanding of AD disease biology [92, 93].

Only one disease-modifying agent has been approved for AD. In 2021, the FDA controversially approved the use of the anti-Aβ monoclonal antibody aducanumab in mild cognitive impairment and early-stage Alzheimer’s disease [94–97]. While both phase III clinical trials of aducanumab were terminated early due to lack of clinical benefit, post hoc analyses revealed one trial met its outcomes of reducing cognitive and functional decline at high aducanumab doses [94–97]. Additionally, both trials revealed a marked decrease in amyloid plaques with high dose aducanumab. Newer monoclonal anti-Aβ antibodies have also shown efficacy in reducing the levels of AD biomarkers in phase II trials, but results are again mixed when considering cognitive and functional benefits [98–100]. Trials of other anti-Aβ or tau therapies have also had mixed results. The γ-secretase inhibitor semagacestat, anti-Aβ monoclonal antibodies bapineuzumab and solanezumab, anti-aggregation agent scyllo-inositol (ELND005), RAGE receptor inhibitor (PF-04494700), and tau aggregation inhibitor TRx0237 have displayed no clinical efficacy in phase III trials of mild-to-moderate AD patients [101–106]. Aβ peptides and tau protein remain strong candidates as therapeutic targets; however, the failure of multiple therapeutic trials highlights the need to consider other targets.

Of note, few anti-inflammatory agents have reached phase III clinical trials to date, although inflammation reflects the second most popular target for current preclinical and clinical AD drug development [92, 93]. The main reason for the delayed development of anti-inflammatory agents is due to conflicting results of epidemiological studies and clinical trial results for nonsteroidal anti-inflammatory drugs (NSAIDs). Multiple epidemiological studies indicate long-term NSAIDs usage reduces AD risk by about 50% in individuals bearing one or more ε4 alleles of apolipoprotein E (apoE), which is strongly associated with increased risk of both familial and sporadic AD [107–112]. Conversely, prospective clinical observations reported traditional NSAIDs or selective cyclooxygenase 2 (COX2) inhibitors did not slow down the cognitive decline associated with mild-to-moderate AD [113–115]. Interestingly, in one large prevention trial, asymptomatic participants showed reduced AD incidence 18–24 months post-NSAID use; however, NSAIDs had adverse effects in patients with cognitive impairment and/or were at a later stage of presymptomatic AD [116]. Taken together, anti-inflammatory agents seem to elicit different effects at various AD stages; anti-inflammatory therapy is beneficial in preventing AD onset but becomes completely non-beneficial in symptomatic AD patients. Counteracting inflammation at later stages by preventing the prolonged microglial and/or astroglial activation during the presymptomatic phase or earlier may be a promising protective strategy.

## G protein-coupled receptors implicated in Alzheimer’s disease

**Fig. 3:**
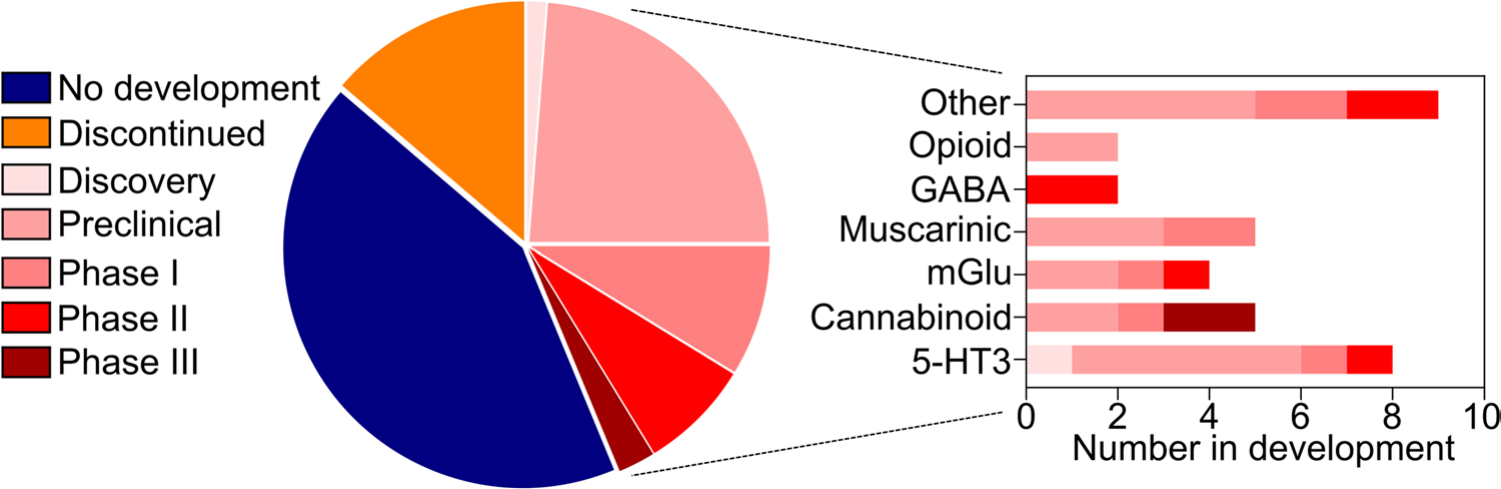
Current status of GPCR-targeted AD therapy development. Of 80 GPCR-targeting agents that have been investigated, 45 have subsequently been discontinued or development halted. Thirty-five are currently in various phases of preclinical and clinical development for AD as both disease-modifying and symptomatic agents, targeting a wide range of GPCR families. “Other” receptors include purinergic (including adenosine), adrenergic, histamine, sphingosine, calcium sensing receptor, gastric inhibitory peptide receptor, glucagon-like peptide 1 receptor, G-protein coupled bile acid receptor 1, and vasoactive intestinal polypeptide receptor. GABA, γ-amino butyric acid; mGlu, metabotropic glutamate. Data compiled using Cortellis Competitive Intelligence software from Clarivate Analytics and is current as of March 2022

Numerous studies have presented compelling results relating GPCRs to AD pathogenesis [117–119]. GPCRs are membrane-bound receptors that transduce external stimuli into signalling cascades within the cell and are important for numerous physiological and pathophysiological processes [120]. Gene expression profiles from AD patients’ postmortem brains via cDNA microarray analysis demonstrate transcript levels of a number of GPCRs changed dramatically, among which were inflammation-associated GPCRs, hormone receptors, and neurotransmitter receptors [121]. Given altered GPCR expression levels would influence the related biological processes, mounting evidence implicates several GPCRs in AD pathogenesis. Several GPCR families have been targeted by putative AD therapies; however, many of these discovery programs have been discontinued (Fig. 3). Of note, there are currently 35 agents targeting GPCRs in the discovery, preclinical, and clinical stages of drug discovery pipelines [122]. Among these are ligands for a range of different GPCR families including serotonergic, cannabinoid, muscarinic, opioid, glutamatergic, and purinergic receptors [122]. Drugs targeting adenosine A_1_ and A_2A_ receptors are of particular interest, owing to the potential role of these receptors in inflammatory processes, as well as both tauopathy and Aβ pathologies [123, 124].

## Adenosine receptors

The adenosine receptors are family A GPCRs, with four structurally similar members; the adenosine A_1_ (A_1_R), the adenosine A_2A_ (A_2A_R), the adenosine A_2B_ (A_2B_R), and the adenosine A_3_ receptor (A_3_R) [125]. Through these receptors, adenosine exerts neuromodulatory effects to regulate essential processes (e.g. neuronal signalling, astrocytic function, learning and memory, motor function, control of sleep and arousal, and normal ageing processes) [126]. Of the four adenosine receptors, A_1_R and A_2A_R show the greatest expression in the brain and have relevance to AD [127, 128], whereas A_2B_R and A_3_R show relatively lower levels of expression [129]. To date, little is known about the role of A_2B_R and A_3_R in AD pathologies.

### Adenosine A_1_ receptor

Mapping A_1_R expression in both the rat and human brain has demonstrated widespread A_1_R distribution, with greater abundance in the hippocampus, cerebral cortex, cerebellum, thalamus, and basal ganglia [130, 131]. In the human cerebellum, A_1_R density is low with strong A_1_R immunoreactivity observed in Purkinje cells. In contrast, in the rat cerebellum, moderate A_1_R expression is detected with weak labelling of Purkinje cells, suggesting these discrepancies in A_1_R expression may reflect species differences. In the rat brain, the highest A_1_R immunoreactivity was found in the large pyramidal neurons of layer 5 of the cerebral cortex and the pyramidal cells in the fields CA2-CA3 of the hippocampus [132]. Furthermore, A_1_Rs are most abundant in synapses, particularly in the presynaptic active zone and postsynaptic density [133, 134]. A_1_Rs are also present in astrocytes, microglia, and oligodendrocytes at a much lower level [135–137].

### Adenosine A_2A_ receptor

Like A_1_R, A_2A_R is also expressed throughout the brain. Highly enriched in the striatum, olfactory tubercle, and nucleus accumbens, A_2A_R is ubiquitously expressed in other brain regions (rat and human) at lower densities [138, 139]. In the rat basal ganglia, A_2A_Rs are predominantly located in dendritic spines and postsynaptic densities, where A_2A_Rs control the integration of signal responses from corticothalamic glutamatergic neurons and medium spiny GABAergic neurons [140, 141]. However, in rat cortical regions, A_2A_Rs are predominantly located in synapses, particularly in the presynaptic active zone [142]. In contrast to A_1_Rs, A_2A_Rs have a broader localisation in different types of nerve terminals. In addition to being expressed in neurons, A_2A_Rs are also located in astrocytes [143, 144] and microglia [145].

## Adenosine A_1_ and A_2A_ receptor signalling

### General signalling

**Fig. 4:**
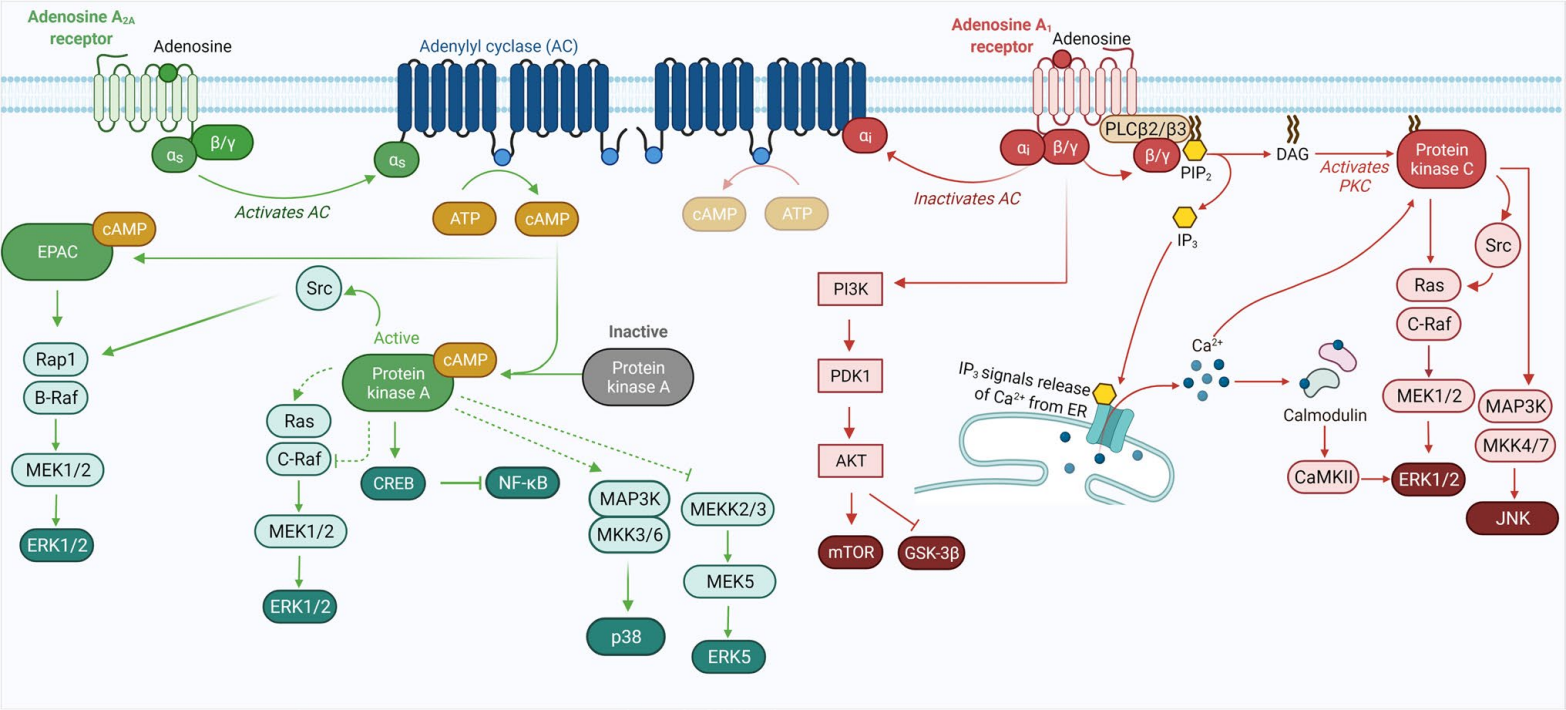
Canonical G_s_ or G_i/o_ signalling pathways upon adenosine A_2A_ and A_1_ receptor activation. Activation of G_s_ stimulates AC, thereby leading to the production of cAMP and PKA stimulation. Gα_s_ elicited the B-Raf-mediated activation of ERKs via Rap-1 or Ras or inhibits C-Raf-mediated activations of ERKs by phosphorylating C-Raf through PKA. Gα_s_ can also stimulate p38 MAPK and inhibits ERK5 via PKA-dependent mechanisms. The dashed lines indicate the pathways remain unclear. In contrast, G_i/o_ inhibits cAMP-dependent signalling and stimulates the activities of ERK, JNK, and PI3K via βγ-subunits-dependent mechanisms. Abbreviations: AC, adenylyl cyclase; AKT, protein kinase B/Akt; ATP, adenosine triphosphate; CaMK, Ca^2+^/calmodulin-dependent protein kinase; cAMP, cyclic adenosine monophosphate; CREB, cAMP response element-binding protein; EPAC, exchange protein directly activated by cAMP; ERK1/2, extracellular signal-regulated kinases 1 and 2; GSK3β, glycogen synthase kinase 3β; JNK, c-Jun N-terminal kinase; MAP3K, mitogen-activated protein kinase kinase kinases; MEK1/2, mitogen-activated protein kinase kinases 1 and 2; mTOR, mammalian target of rapamycin; NF-κB, nuclear factor kappa-light-chain-enhancer of activated B cells; PDK1, 3-phosphoinositide dependent protein kinase-1; PI3K, phosphoinositide 3 kinase; PKA, protein kinase A; PLC, phospholipase C; Src, proto-oncogene tyrosine-protein kinase. Created with BioRender.com

Signal transduction mediated by A_1_R and A_2A_R is largely driven by coupling to heterotrimeric G protein complexes, which are composed of a Gα subunit and Gβγ heterodimeric complex (Fig. 4) [146]. Pertussis toxin-sensitive G_i/o_ proteins are preferentially activated by A_1_R, whereas A_2A_R shows a preference for G_s_ and G_olf_ proteins, with the latter being primarily restricted to striatal brain regions [147–149]. These G proteins affect the activity of adenylyl cyclase (AC), with Gα_s_ proteins activating AC and increasing the production of the second messenger cyclic adenosine monophosphate (cAMP) from ATP [150]. Conversely, stimulation of Gα_i/o_ proteins results in AC inhibition and thereby reduces cAMP production. Downstream targets of cAMP include cyclic nucleotide-gated ion channels (HCN), the small G protein guanine nucleotide exchange factor (EPAC) and protein kinase A (PKA), all of which play important roles in neural physiology [151, 152].

In addition to cAMP signalling, both A_1_R and A_2A_R also signal via additional effectors, including mitogen-activated protein kinases (MAPK) and Akt/protein kinase B, which play a role in cell growth, survival, differentiation, and protein transcription and translation [153, 154]. A_1_R stimulates the extracellular signal-regulated kinases 1 and 2 (ERK1/2), c-Jun N-terminal kinases (JNK), and the p38 MAPK, and additionally phosphorylates Akt via phosphoinositide 3-kinase (PI3K) activation [155–162]. This signalling is largely driven via interactions of βγ subunits with effectors upon dissociation from Gα_i/o_ proteins. Additionally, A_1_R mobilises intracellular calcium stores and activates multiple isoforms of protein kinase C, via direct βγ interactions with phospholipase C [162–165]. Similarly, A_2A_R also signals via ERK1/2, JNK, p38, and Akt, with these effects thought to be downstream of α subunit activity of G_s_ [156, 166–173]. Interestingly, some studies support a stimulatory or inhibitory effect of these second messengers by A_2A_R, suggesting this signalling can be conditional, depending on cell background and disease context [174, 175].

### Neuronal signalling

In addition to the above-generalised signalling pathways, A_1_R and A_2A_R both stimulate additional pathways and effectors that are either brain-specific or present in only niche cell types. A_2A_R has been established as a modulator of neurotrophins, including brain-derived neurotrophic factor (BDNF) and nerve growth factor (NGF), which play important roles in neuronal differentiation and survival, in addition to regulating synaptic transmission and plasticity [176]. In microglia and hippocampal slices, A_2A_R can increase the release of both BDNF and NGF, which may be mediated by the transactivation of neurotrophic receptors [177–181]. A_1_R has also been suggested to play a role in neurotrophin signalling, although its effects are less well defined [182].

Modulation of ion channel activity represents another vital signalling mechanism for both A_1_R and A_2A_R. A_1_R couples to a number of ion channels mediating an overall net hyperpolarisation of neurons [183]. It is well established that A_1_R couples to G protein-coupled inwardly-rectifying potassium (GIRK) channels, which are a class of transmembrane proteins that facilitate potassium flux into the cytosol [184–186]. A_1_R coupling to GIRK channels is through the direct binding of βγ subunits. Additionally, A_1_R reduces the current of voltage-dependent calcium channels, with a proposed preference for N-type channels [187–190]. Alongside A_1_R, A_2A_R also couples to voltage-dependent calcium channels; however, A_2A_R has a stimulatory effect on the current [187, 191]. Both receptors can also activate ATP-sensitive potassium channels (K_ATP_), which are present in plasma, mitochondrial, and nuclear membranes, and play a role in neuronal excitability and survival [192, 193].

Collectively, these various signalling streams result in an overall inhibitory effect of A_1_Rs and excitatory effects of A_2A_Rs, allowing fine-tuning of neuronal circuitry. Activation of presynaptic A_1_Rs on excitatory neurons reduces neurotransmitter release and induces synaptic depression, whereas presynaptic A_2A_Rs are involved in increasing neurotransmitter release [133, 194]. Similarly, postsynaptic A_1_R activation causes membrane hyperpolarisation with subsequent inhibition of neuronal firing. In contrast, activated postsynaptic A_2A_Rs increase cellular excitability [195]. Importantly, the effects of both A_1_R and A_2A_R are not limited to the modulation of neuronal activity, these receptors also coordinate the function of additional cells, including astrocytes and microglia (see [196] for review). These effects impart additional fine-tuning of neural homeostasis and inflammatory balance.

## The role of A_1_R and A_2A_R in dementia

Animal models of AD and old age, alongside human postmortem analyses, have revealed evidence of a disruption to the neural adenosine network [197, 198]. Levels of adenosine and its related metabolites are altered in the brains of patients with AD [199, 200]. Similarly, adenosine receptors (AR) change their pattern of localisation and density in affected brain regions of AD [201–203]. Postmortem analysis of AD patients’ brains showed reduced A_1_R expression at the dentate gyrus and hippocampal CA3 regions [204], which are focal points for the spread of NFTs and subsequent neuronal loss [44]. PET studies with a radiolabelled A_1_R antagonist have also demonstrated reduced A_1_R levels in the temporal cortex in patients with AD compared to elderly subjects [205]. Moreover, postmortem AD frontal cortex samples showed increased A_1_R and A_2A_R expression, compared to age-matched controls [202]. Analysis of patients with frontotemporal lobar degeneration revealed increased A_2A_R expression in the temporal cortex and enhanced A_2A_R immunoreactivity in neurons expressing tau pathology [206]. Moreover, A_2A_R expression is increased in the aged forebrain when compared to young subjects, with a further significant increase in AD patients [207]. Significantly increased brain A_2A_R levels in AD patients are coupled with peripheral platelets also reflecting an increase in A_2A_R [208]. Accordingly, peripheral A_2A_R expression could act as a biomarker for dementia and inform disease progression.

Animal and cellular models recapitulate these changes to AR expression, with increased A_1_R immunoreactivity in neurons with NFTs and in amyloid plaques, alongside enhanced glial A_2A_R expression [201]. In human neuroblastoma cells and primary rat cortical neurons, administration of Aβ_25-35_ increased A_1_R and A_1_R/A_2A_R expression, respectively [209, 210]. APP/PS1 AD mice show increased A_1_R and A_2A_R levels compared to non-transgenic mice, and rats with sporadic dementia also show elevated A_2A_R levels [123, 211, 212]. The 5XFAD model of AD also demonstrates increased A_1_R expression, highlighting changes to ARs can be found across different animal models, suggesting a central role in the pathogenesis of dementia [209]. Overall, the precise mechanisms underlying the dysregulation of A_1_R and A_2A_R in AD/dementia remain unresolved. However, it has been suggested that early in the disease process, disruption to the homeostatic levels of adenosine may impart dysfunctional regulatory feedback, which modifies the expression and locality of AR receptors [200].

Epidemiological studies have reported an inverse association between caffeine intake and AD/dementia risk [213–217]. Significant caffeine consumption is associated with a lowered rate of Aβ positivity as measured by PET [218]. Indeed, caffeine, acting as a nonselective A_1_R and A_2A_R receptor antagonist, reduces Aβ toxicity and enhances cognition in numerous models. The neuroprotective qualities of caffeine have been demonstrated in cultured rat and mouse neurons, where Aβ-induced neurotoxicity and tau phosphorylation was reduced, respectively [123, 219]. In APPsw transgenic mice and a rat model of sporadic dementia, caffeine promoted neuroprotection and mitigated cognitive impairment [123, 212]. Long-term administration of caffeine to AD transgenic mice also improved cognition and reduced Aβ_o_ generation and was accompanied by a modest reduction in presenilin-1 and β-secretase expression levels [220]. These neuroprotective actions have largely been ascribed to caffeine’s A_2A_R antagonism, a notion supported by studies using selective A_2A_R antagonists (e.g. KW6002, SCH58261, ZM241385, MSX-3). In animal models, including APP/PS1 mice and THY-Tau22 mice, administering selective A_2A_R antagonists improved memory deficits and mitigated the Aβ toxicity or tau hyperphosphorylation associated with the disease [207, 211, 221]. Moreover, the administration of human Aβ_1-42_ fragment reduced memory performance in rats, which was reversed by SCH58261 and KW6002 [222]. Interestingly, in this model, A_2A_R antagonism could not mitigate acute memory deficits induced by the muscarinic receptor antagonist scopolamine or the NMDA antagonist MK801. As such, it has been suggested the beneficial effects of A_2A_R antagonists on memory may not be a generalised effect but rather specific to the disease processes involved in dementia/AD.

Alongside animal studies, pharmacological A_2A_R blockage in neurons attenuated Aβ-induced neuronal death and further reduced synaptic loss [223]. Similarly, in cultured rat cerebellar neurons ZM241385 was neuroprotective; however, A_1_R antagonist CPX was ineffective, implying Aβ-induced neurotoxicity is primarily mediated through A_2A_R activity [224]. Additional studies have provided diverging roles for A_1_R. In a model using human neural cells, application of the selective A_1_R agonist R-PIA led to soluble APP production and increased tau phosphorylation, which was reversed by A_1_R-selective antagonist DPCPX [201]. In tau transgenic mice, A_1_R antagonist rolofylline restored memory deficits and reduced synaptic dysfunction in neural cells [225]. Given that A_1_R redistributes and co-localises with NFTs and Aβ plagues in AD patients, these studies suggest A_1_R may play a direct role in mediating some of the pathology [201, 202]. Importantly, in addition to pathological signalling, A_1_R activation is strongly neuroprotective in a number of settings [226] and elicits anti-inflammatory effects in chronic neuroinflammation [124, 227]. Indeed, acute administration of an A_1_R agonist decreased neurodegeneration in in vitro and in vivo models challenged with noxious stimuli [227]. As such, selective A_1_R antagonists may protect against β-amyloid and tau neurotoxicity and enhance cognition, whereas A_1_R agonists may impart neuroprotection.

In addition to pharmacological studies, A_2A_R genetic deletion or overexpression has also revealed interesting findings. In APP/PS1 mice, A_2A_R downregulation via shRNA restored long-term potentiation and improved memory deficits [211]. In contrast, A_2A_R overexpression in cortical and hippocampal neurons of rats resulted in increased glutamate release, which was associated with changes in synaptic plasticity [207]. In THY-Tau22 mice, genetic deletion of A_2A_R protected against spatial memory deficits, reduced neuroinflammation, and decreased tau hyperphosphorylation [221]. Conversely, selective A_2A_R overexpression in the forebrain of THY-Tau22 mice resulted in tau hyperphosphorylation and increased memory deficits [206]. Mice also showed increased expression of hippocampal c1q complement protein, a biomarker found in patients with frontotemporal lobar degeneration, suggesting A_2A_R may contribute to this process. Interestingly, upregulation of genes associated with immune responses was also found in this study, with further cell-specific enrichment analysis revealing these genes were preferentially increased in microglia. As such, microglial A_2A_R and its associated immune responses may play a role in the pathogenesis of dementia. Indeed, additional studies support the importance of glial cells, with A_2A_R being upregulated in microglia and astrocytes after treatment with Aβ_o_ [228] and conditional genetic ablation of astroglial A_2A_R enhancing long-term memory in young and in ageing mice [229]. Targeting the NLRP3 (nucleotide-binding oligomerisation domain-, leucine-rich repeat-, and pyrin domain-containing 3) inflammasome to modulate AD pathology has increasing interest as a therapeutic strategy [230], with emerging evidence of A_2A_R in microglia offers an upstream therapeutic target [231]. A_2A_R activation stimulates sustained NLRP3 inflammasome activity and the production of proinflammatory cytokines (e.g. IL-1β) in macrophages and primary microglia [232–235]. Notably, in preclinical models of hypoxic-ischemia and autoimmune encephalomyelitis, caffeine inhibited NLRP3 inflammasome activation and microglial activation to confer neuroprotection and attenuate disease pathology [236, 237]. Therefore, in addition to modulating neuronal signalling, targeting A_2A_R on microglia may present an opportunity to target neuroinflammation associated with AD.

## Therapeutic paradigms for targeting adenosine receptors

### A_1_R and A_2A_R: antagonism or agonism?

The growing evidence supporting a role for A_1_R and A_2A_R in dementia/AD highlights these GPCRs represent promising drug targets. Overall, the studies described predominantly indicate that A_1_R and A_2A_R antagonism may be a viable therapeutic approach to mitigate pathology and improve patient symptoms. Indeed, A_1_R antagonism may afford protection against β-amyloid and tau neurotoxicity and the enhancement of cognition is clearly desirable in the setting of dementia. However, the neuroprotective nature of A_1_R is also an important consideration. Studies showing reduced brain A_1_R expression in patients with AD make this particularly pertinent, as reduced A_1_R expression could facilitate neuronal excitotoxicity [238]. As such, it is interesting to speculate how the administration of an agonist or antagonist would fare clinically, given the divergent preclinical data. Similarly, the protection afforded by A_2A_R antagonism against β-amyloid neurotoxicity and memory impairment in preclinical settings makes it a desirable drug target. The A_2A_R antagonist istradefylline enhanced cognition in mice with amyloid pathology and is a clinically used adjunctive therapy in Parkinson’s disease [239, 240]. Istradefylline has not been tested in humans with AD/dementia; however, this may represent a worthwhile exploratory investigation given this compound is clinically available in some countries. Interestingly, A_2A_R agonism may be useful very early in the disease process, where its anti-inflammatory effects may dampen disease progression [241]. A_2A_R induction of BDNF signalling could also contribute to greater neuronal survival and maintenance of synaptic plasticity; however, these considerations remain largely unexplored.

Nonetheless, despite the promising preclinical data, the void of clinically approved drugs targeting A_1_R or A_2A_R for AD/dementias remains apparent. This may be partly owing to the need for a deeper understanding of the precise roles of A_1_R and A_2A_R in AD/dementia. Moreover, the widespread distribution of ARs and their role in fundamental biological processes is an additional hurdle for the drug discovery process. For example, A_1_R activation is associated with lowered heart rate and altered blood pressure [242], whilst antagonism is associated with increased seizure risk and sleep disturbance [243, 244]. A_2A_R antagonism, such as that mediated by istradefylline, can cause involuntary movements and hallucinations [245]. Many of these on-target side effects are centrally driven, making it difficult to dissociate the therapeutic and adverse effects. As such, prototypical AR agonists or antagonists may encounter challenges during clinical translation. Alternative pharmacological approaches that circumvent these limitations can minimise the risk of on-target adverse effects.

### Allosterism

Allosteric ligands may overcome many of the limitations associated with traditional agonists and antagonists. Allosteric compounds bind to a topographically distinct site acting to modulate the affinity and/or efficacy of the orthosteric endogenous ligand, in this case, adenosine [246]. A larger number of pharmacological parameters drive the activity of allosteric ligands when compared with orthosteric compounds, which affords more nuanced signalling. This is evidenced by the broad categories in which they can be classed, including (i) allosteric agonists, which increase (agonist) or decrease (inverse agonist) signalling by binding to the allosteric site; (ii) positive or negative allosteric modulators (PAM or NAM), which can increase or decrease, respectively, the affinity and/or efficacy of an orthosteric ligand; and (iii) neutral allosteric ligands (NAL), which exhibit neutral cooperativity with the orthosteric ligand and have no intrinsic efficacy in their own right [247]. Moreover, the pharmacological parameters encoded by allosteric ligands can be fine-tuned by medicinal chemistry and structure-based efforts. As such, allosteric modulators belonging to the same class (PAM, NAM, or allosteric agonist) can elicit a broad spectrum of activity and thus can be rationally selected based on the underlying disease.

Allosteric ligands offer numerous advantages. Since allosteric sites typically show greater sequence divergence compared to orthosteric sites, allosteric ligands typically exhibit greater subtype selectivity [246, 248]. As such, allosteric ligands can impart efficacy and/or affinity modulation upon endogenous adenosine in a subtype-selective manner. Additionally, allosteric modulators can signal in a spatiotemporally specific manner, modifying the signalling of adenosine only where and when it is present [246, 249]. This feature is especially important in AD/dementia, as evidence suggests adenosine levels in the brain can vary in a region-specific manner [199].

### Therapeutic potential of A_1_R and A_2A_R allostery

Allostery has been detected and quantified at both A_1_R and A_2A_R, although there has been significantly more traction at A_1_R. A large number of A_1_R PAMs have been discovered, with very early studies characterising the allosteric enhancer PD 81,723 [250]. The utility of A_1_R PAMs has been demonstrated preclinically and in a phase II trial, confirming this pharmacological approach has the translational potential [249, 251]. To date, selective A_1_R NAMs have not been identified. A_2A_R allosteric modulators are limited. A fragment-based drug discovery approach identified potential PAM and NAM scaffolds for A_2A_R [251–253]. These findings, along with recent structural advances in the AR field [254–257], are likely to accelerate the discovery of new A_1_R and A_2A_R allosteric ligands. Despite the paucity of A_1_R and A_2A_R NAMs and the lack of evaluation of these classes of compounds in AD models, the physiological advantages remain conceptually promising. For A_1_R and A_2A_R NAMs, inhibiting signalling only where there is increased adenosine tone could mitigate β-amyloid neurotoxicity and enhance cognition, whilst avoiding the risk of seizures, sleep, and motor disturbances commonly associated with antagonism. This could afford a much more nuanced fine-tuning of AR signalling, without globally reducing adenosine function across all brain areas. Furthermore, this would also likely reduce the incidence of peripheral cardiovascular side effects.

### Receptor oligomerisation (homo- and heteromerization)

Another form of allostery to consider when targeting A_1_R and A_2A_R is protein–protein interactions. The traditional model of GPCR activity depicted the receptors to function exclusively as monomeric entities. Over the last two decades, increasing evidence indicates GPCRs form homomers and heteromers or higher-order oligomers as part of GPCR normal trafficking and function [258, 259]. Homomerisation describes the self-association of receptor subunits, while heteromerisation describes the association of two or more different receptor subunits, with biochemical properties demonstrably different from individual components. Recent studies support the existence of A_1_R homomers at the plasma membrane using bimolecular fluorescence complementation and fluorescence correlation spectroscopy [260]. Conversely, cell surface A_2A_R homomers were confirmed in bioluminescence resonance energy transfer (BRET) or Förster resonance energy transfer (FRET) experiments [261] and may form into oligomers with three or more A_2A_R protomers [262]. Heteromers and/or higher-order oligomers between different AR subtypes (for review see [263]), as well as with unrelated GPCRs receptors and signalling complexes with other membrane proteins in the brain have increased attention. For an oligomeric interaction to be considered physiologically significant, it is critical to show physical association in native tissue or primary cells and demonstrate unique ‘biochemical fingerprints’ distinct to the oligomer [264].

There are two general mechanisms by which receptor oligomerisation can influence the drug effect [265]. (1) The receptor oligomer becomes a new conduit, whereby ligand-oligomer interactions generate a unique biochemical signalling fingerprint. For example, A_2A_R agonists decrease the affinity and intrinsic efficacy of D_2_R agonists in A_2A_R-D_2_R heteromers [266]. (2) One receptor modulates ligand binding and/or signalling effects mediated by the other receptor. For example, D_2_R selectively confers negative cooperativity towards the A_2A_R antagonist SCH442416 in a A_2A_R-D_2_R heteromer, compared to when not forming heteromers or forming heteromers with A_1_R [267]. As such, SCH442416 would less effectively target A_2A_R-D_2_R heteromers expressed in striatopallidal neurons, compared to presynaptic A_2A_R-A_1_R heteromers localised in cortico-striatal glutamatergic neurons. Heteromers/homomers with allosteric properties provide an exciting possibility to fine-tune receptor signalling, trafficking, and pharmacological properties.

### Therapeutic potential of targeting AR oligomers

Receptor oligomerisation gives rise to novel therapeutic interventions, such as co-activation or co-inhibition of both protomers and activation of one protomer while inhibiting another protomer. Targeting A_2A_R-D_2_R heteromers is a well-established therapeutic strategy for Parkinson’s disease where A_2A_R antagonism in conjunction with D_2_R agonism is highly desired. Commonly, A_2A_R antagonists are dosed in conjunction with L-DOPA; however, there are continuing efforts to develop bivalent ligands to co-occupy and specifically target A_2A_R-D_2_R heteromers [268–270]. Beyond A_2A_R-D_2_R heteromers, multiple bona fide AR heteromeric complexes are of therapeutic interest and relevant in the setting of AD (Table 1). A number of recent reviews provide in-depth coverage of the scope of AR heteromerisation [263, 271, 272].

**Table 1:** Selected examples of AR heteromers and summary of evidence for in vitro and in vivo physical interactions and functional effects

Heteromer	In vitro-heterologous expression				In vivo-native cells/tissue				Criteria fulfilled for native heteromers^a^	References
	Co-loc	Co-IP	PBA	Unique properties	Co-loc	Co-IP	PBA	Unique properties		
A_1_R-A_2A_R	✓	✓	✓	✓	✓	✓	n.d	✓	2	[273, 274]
A_1_R-mGlu_1a_	✓	✓	n.d	✓	✓	✓	n.d	✓	2	[281, 282]
A_1_R-P2Y_1_R	✓	✓	n.d	✓	✓	✓	n.d	✓	2	[283–286]
A_2A_R-A_3_R	n.d	n.d	✓	✓	n.d	n.d	✓	✓	2	[275, 287]
A_2A_R-mGlu_5_	✓	✓	✓	✓	✓	✓	✓	✓	1,2	[141, 288–292]
A_2A_R-D_2_R	✓	✓	✓	✓	✓	✓	✓	✓	1,2,3	[270, 293–298]
A_2A_R-CB_1_R	✓	n.d	✓	n.d	✓	✓	✓	✓	1,2	[299–303]
A_2A_R-NMDAR	✓	n.d	✓	✓	n.d	n.d	✓	✓	2	[278]

✓, demonstrated; *AR*, adenosine receptor; *CB*, cannabinoid receptor; *Co-loc*, co-localisation; *Co-IP*, co-immunoprecipitation; *D*_*2*_*R*, dopamine D2 receptor; *mGlu*, metabotropic glutamate receptor; *n.d,* not determined; *NMDAR*, N-methyl-D-aspartate ionotropic glutamate receptor; *P2Y*_*1*_*R*, purinergic P2Y_1_ receptors; *PBA*, proximity-based assays

^a^Criterion 1: Heteromers exhibit appropriate colocalisation and interaction to enable allosterism (considered fulfilled if there is evidence from proximity-based assays, colocalisation and coimmunoprecipitation). Criterion 2: Heteromers exhibit distinct properties. Criterion 3: Heteromer-selective reagents alter heteromer properties

As discussed above, A_1_R and A_2A_R are promising therapeutic targets for AD. A_1_R-A_2A_R heteromers are expressed in neurons and glia, with heteromers modulating neurotransmitter levels in the context of a tripartite synapse [273, 274]. A_1_R-A_2A_R heteromers can be considered a ‘thermostat’ for extracellular adenosine levels, with the opposing effects of G_i_ versus G_s_ coupling controlling brain cell responses depending on whether adenosine levels are high or low. Therefore, receptor oligomerisation may also raise unexpected confounds and impacts due to altering the signalling balance of heteromers. Further, oligomerisation may be cell type- or brain region-specific. For example, recent reports of A_2A_R-A_3_R heteromers show differential brain-region expression, being the highest in striatal neurons compared to the hippocampus or frontal cortex but are also found in microglia [275]. To therapeutically exploit A_2A_R-A_3_R, it would be desirable to inhibit A_2A_R, thereby removing A_2A_R functional antagonism of A_3_R signalling and shifting the balance towards A_3_R activation by endogenous adenosine. Despite being found at low levels in the brain, A_3_R is suggested to be neuroprotective in a number of settings, including traumatic brain injury and cerebral ischemia [276, 277]. Targeting AR oligomers linked to disease biology also offers the potential for greater selectivity. Proximity-based approaches suggest heterocomplexes between A_2A_R and N-methyl-D-aspartate ionotropic glutamate receptors (NMDAR) are increased in activated microglia as well as in the hippocampus of transgenic AD mice (APP_sw, Ind_) [278]. A_2A_R has a central role in modulating mGlu_5_, D_1_R, and NMDAR signalling in the hippocampus under physiological conditions [279], possibly linked to the propensity of A_2A_R to form heteromeric complexes.

## Concluding remarks

Dementia remains a significant neurological disorder. This review has highlighted preclinical studies implicating A_1_R and A_2A_R as promising GPCR targets for Alzheimer’s disease. Overall, A_1_R and A_2A_R inhibition seems to mitigate the neurotoxicity associated with the β-amyloid accumulation and tau hyperphosphorylation and improve cognition and memory. Stimulation of A_1_R can also promote neuroprotection and therefore adds an additional layer of complexity in considering agonism versus antagonism when targeting this receptor. Although not explored for AD, the notion of biased agonism, which is a growing paradigm at adenosine receptors [249, 280], could conceptually be harnessed to develop an A_1_R compound to improve cognition but also remain neuroprotective. Indeed, although drug discovery at adenosine receptors has traditionally experienced hurdles, the novel therapeutic paradigms covered in this review, including allostery and the targeting of oligomers, present a promising future avenue of investigation. It is hoped harnessing this knowledge may increase the development and translation of clinical candidates with enhanced therapeutic responses and limited side effects.

## Data Availability

Not applicable.
