# Publisher Correction to: Adenosine receptor signalling in Alzheimer’s disease

**DOI:** 10.1007/s11302-022-09906-x

**Published:** 2022-11-16

**Authors:** Phuc N. H. Trinh, Jo-Anne Baltos, Shane D. Hellyer, Lauren T. May, Karen J. Gregory

**Affiliations:** 1 Drug Discovery Biology, Monash Institute of Pharmaceutical Sciences, Monash University, Parkville, VIC 3052 Australia; 2 Department of Pharmacology, Monash University, Parkville, VIC 3052 Australia; 3 ARC Centre for Cryo-Electron Microscopy of Membrane Proteins, Monash Institute of Pharmaceutical Sciences, Parkville, 3052 Australia

**Abstract**

Alzheimer’s disease (AD) is the most common dementia in the elderly and its increasing prevalence presents treatment challenges. Despite a better understanding of the disease, the current mainstay of treatment cannot modify pathogenesis or effectively address the associated cognitive and memory deficits. Emerging evidence suggests adenosine G protein-coupled receptors (GPCRs) are promising therapeutic targets for Alzheimer’s disease. The adenosine A1 and A2A receptors are expressed in the human brain and have a proposed involvement in the pathogenesis of dementia. Targeting these receptors preclinically can mitigate pathogenic β-amyloid and tau neurotoxicity whilst improving cognition and memory. In this review, we provide an accessible summary of the literature on Alzheimer’s disease and the therapeutic potential of A1 and A2A receptors. Although there are no available medicines targeting these receptors approved for treating dementia, we provide insights into some novel strategies, including allosterism and the targeting of oligomers, which may increase drug discovery success and enhance the therapeutic response.

This article is part of the Special Issue “Puringergic Signalling – Perspectives from Australia and New Zealand”, Guest Editors: Ronald Sluyter, School of Chemistry and Molecular Bioscience, University of Wollongong, Australia; Jennie Cederholm, School of Medical Sciences (SoMS), UNSW Sydney, Australia; Srdjan Vlajkovic, Dept. of Physiology, University of Auckland, New Zealand;

It was unintentionally published in issue 18, 359–381 (2022). You can access the article via this link: https://link.springer.com/article/10.1007/s11302-022-09883-1. We apologise for the inconvenience.

